# A Simple Artificial Life Model Explains Irrational Behavior in Human Decision-Making

**DOI:** 10.1371/journal.pone.0034371

**Published:** 2012-05-01

**Authors:** Carolina Feher da Silva, Marcus Vinícius Chrysóstomo Baldo

**Affiliations:** “Roberto Vieira” Laboratory of Sensory Physiology, Department of Physiology and Biophysics, Institute of Biomedical Sciences, University of São Paulo, São Paulo, Brazil; National Research & Technology Council, Argentina

## Abstract

Although praised for their rationality, humans often make poor decisions, even in simple situations. In the repeated binary choice experiment, an individual has to choose repeatedly between the same two alternatives, where a reward is assigned to one of them with fixed probability. The optimal strategy is to perseverate with choosing the alternative with the best expected return. Whereas many species perseverate, humans tend to match the frequencies of their choices to the frequencies of the alternatives, a sub-optimal strategy known as *probability matching.* Our goal was to find the primary cognitive constraints under which a set of simple evolutionary rules can lead to such contrasting behaviors. We simulated the evolution of artificial populations, wherein the fitness of each animat (artificial animal) depended on its ability to predict the next element of a sequence made up of a repeating binary string of varying size. When the string was short relative to the animats’ neural capacity, they could learn it and correctly predict the next element of the sequence. When it was long, they could not learn it, turning to the next best option: to perseverate. Animats from the last generation then performed the task of predicting the next element of a non-periodical binary sequence. We found that, whereas animats with smaller neural capacity kept perseverating with the best alternative as before, animats with larger neural capacity, which had previously been able to learn the pattern of repeating strings, adopted probability matching, being outperformed by the perseverating animats. Our results demonstrate how the ability to make predictions in an environment endowed with regular patterns may lead to probability matching under less structured conditions. They point to probability matching as a likely by-product of adaptive cognitive strategies that were crucial in human evolution, but may lead to sub-optimal performances in other environments.

## Introduction

In economics, politics, and the social sciences, it is often assumed that humans make rational decisions, especially in simple situations that repeat themselves [1]. The so-called *rational choice* theory models human beings as agents who go about achieving their self-interested goals in the best possible way, maximizing their expected utility [2]. This theory, often in conjunction with game theory, is used to predict the behavior of individuals.

Yet consider a simple decision making task, wherein a subject has to choose between two alternatives – a binary choice problem. When asked to predict the next element in a sequence of coin tosses, for instance, many people believe that the chance of getting a tail increases after several heads in a row [3]. This belief, known as the gambler’s fallacy, is incorrect and may lead to sub-optimal performance.

Similarly, in a different task widely studied since the 1940’s [1] and known as the *repeated binary choice experiment*, an individual has to choose between two repeatedly presented alternatives and a reward is randomly associated, with probability greater than 0.5 and lesser than 1, to one of the alternatives. For instance, at each trial, a light may flash either on the left or on the right of a screen and the subject is asked to predict which side the light will flash and is rewarded if the prediction is correct. The side where the light will actually appear is chosen by a computer program independently at each trial, with a constant probability for each side; for instance, the light may flash on the left with 2/3 (67%) probability and on the right with 1/3 (33%) probability. This makes it impossible to predict correctly all the time where the light will flash. Instead, once the subject has realized the light flashes on one side (the *majority* side) more frequently than on the other (the *minority* side), the optimal strategy is to perseverate with choosing the majority side. This strategy is called *perseveration*, and subjects that perseverate will be correct on about two thirds (67%) of the trials, which is the best anyone can do.

Human adults, however, don’t perseverate as a rule [1]. They tend to choose a given side with about the same frequency with which the light is flashed on that side. This strategy, known as *probability matching*, is sub-optimal: in the previous example, subjects that employ probability matching will be correct only in about five ninths (56%) of the trials ((1/3)^2^+(2/3)^2^), one ninth (11%) below perseveration (2/3 = 6/9). Surprisingly, other animals such as rats [4], monkeys [5], pigeons and fish [6] tend to perseverate, maximizing their returns. Thus, in a repeated binary choice experiment, human beings not only do not maximize their expected utility, but they are also outperformed by rats and fish.

It has been previously suggested that human subjects do probability matching because they look for patterns or rules that might be determining the sequence of outcomes [1], [7]. Indeed, when asked to describe what strategy they employed in the experiment, they have themselves reported that they look for patterns. In searching for a putative underlying pattern, humans might be playing around with different alternatives, eventually doing probability matching. However, evidence supporting this view is sparse and indirect [8]–[14] and alternative explanations have proliferated, such as mistaken mathematical intuition [15], insufficient motivation and practice [16], adaptation to uncertainty [17], [18], adaptation to foraging in a competitive environment [19], [20], and probability matching as a consequence of nearly optimal structure learning [21].

It could also be questioned how a sub-optimal behavior could arise from evolutionary constraints favoring the selection of cognitive resources capable of detecting regularities in a seemingly unruly world. Given our evolved cognitive apparatus, instead of being trapped in a maladaptive behavior, we should be able to switch to the maximizing strategy and perseverate with the most frequently rewarding alternative as soon as the unfeasibility of finding any regular underlying pattern had been detected.

Here we propose an artificial life model that helps us understand how being selected for learning structured patterns may lead to probability matching, and how failure to learn them leads to perseveration. Also, we demonstrate that the cognitive ability to detect underlying patterns in a scenario of prevailing regularities can compensate, in an evolutionary sense, for the sub-optimal performance that results from the persistence in seeking for patterns when none are present.

## Results

In our simulations, artificial animals (*animats*) had to perform a task, called the *pattern matching task.* It consisted of predicting repeatedly the next element of a binary sequence formed by a repeating string. An example string is 101, leading to the sequence 101101101… During their lives, at each time-step, animats had to choose between 0 and 1. When their choices matched the next element of the sequence, they won fitness points. The length of the repeating string, and thus its difficulty to be learned, varied in different simulations among 3, 9, 27, 81, 243 or 729 digits (six different lengths). The frequencies of the digits 0 and 1 in the repeating strings were always 1/3 and 2/3 respectively, and the strings were repeated until total sequence length was 2916. We tested twelve randomly generated strings of each length, and each was individually presented to a group of 100 animats in separate simulations, adding up to a total of 144 evolving scenarios (6 lengths × 12 permutations × 2 neural network architectures), taking into account the two kinds of neural network architectures employed in the simulations (see below). Although there are only three possible strings of length 3 (011, 101 and 110), we still ran twelve simulations by repeating four times each of the three possible patterns.

**Figure 1:**
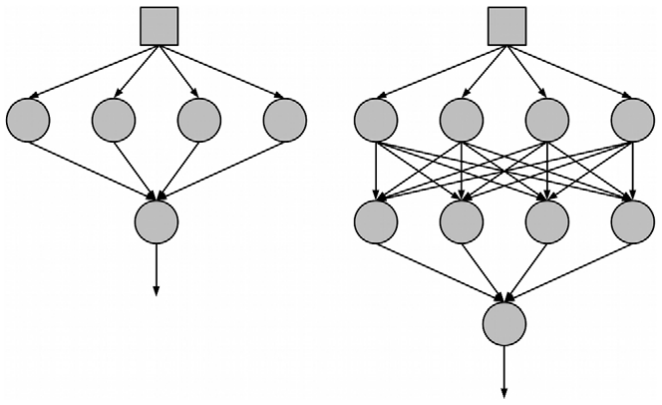
Neural Network Architectures Used in the Simulations. Two different neural network architectures were used in the simulations. Networks had one input node, one or two layers of four hidden nodes, and one output node.

Animats were endowed with neural networks to model a simple nervous system. Artificial neurons were based on a simple model of biological neurons, the *perceptron* [22], and were connected into neural networks with one input node, one or two hidden layers of four nodes, totaling four or eight hidden nodes, and one output node (Figure 1). The number of hidden nodes correlates with computational power, with these networks exhibiting lower (4 nodes) or higher (8 nodes) learning potentials.

Depending on the network output, we considered that the animats had predicted the next element of the sequence to be 0 or 1. The input node delivered a feedback signal from the environment to the neural network at time *t* about the animat’s response at time *t − 1* (see *Methods* for details), analogous to the feedback received by subjects in a repeated binary choice experiment (usually a message on a computer screen – “You won!” or “You lost.” – or a reward). The synaptic weights, which measure the strength of the synapses between neurons, could change through Hebbian learning, thus enabling the animat to have different responses to the same stimuli depending on the previous state of the network, which is necessary for the network to repeat the patterns.

Populations of animats evolved through a genetic algorithm, which models biological evolution according to a simplified version of Darwin’s theory. At generation zero, one hundred chromosomes were generated randomly (a chromosome was a set of genes representing all the synaptic weights, biases and learning parameters – see *Methods* for details). Neural networks were constructed based on these chromosomes and the animats performed the pattern matching task described above. The number of correct answers was the fitness value, a measure of evolutionary fitness and performance in the task. Then a new set of animats – the next generation – was created through selection, mutation and crossover. The populations evolved for 1000 generations.

The animats from the last generation performed an additional task, which we called the *random sequence task.* It was similar to the pattern matching task in that the animats had to predict the next element of a binary sequence of length 2916, but the binary sequence was no longer formed by a repeating string. Instead, its elements were randomly shuffled, destroying any regularity but keeping unchanged the digit frequencies that characterized the repeating string sequences previously employed. Performance in this task was compared to the performance in the pattern matching task for all animats from the last generation of all the simulations by calculating the prediction accuracy (the ratio of the number of correct predictions to the total number of predictions) and average response (or average prediction). Thus, we could test if animats with more neurons, which were able to learn longer patterns, were also more prone to do probability matching when confronted with random sequences.

**Figure 2:**
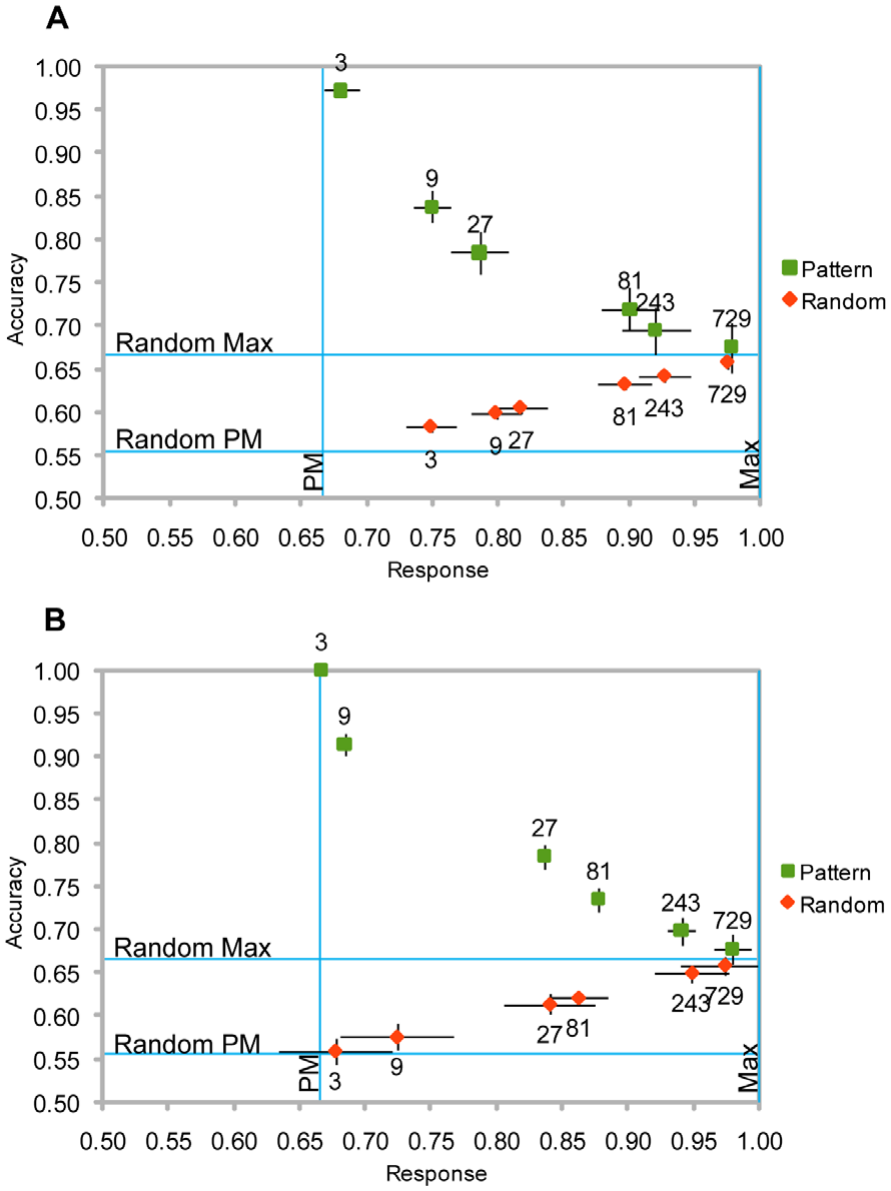
Prediction Accuracy and Average Response for Different Pattern Lengths in Both Tasks (Pattern, Random). The prediction accuracy and average response for different pattern lengths in both tasks: *Pattern*, when the sequence was formed by a repeating pattern, and *Random*, when the sequence was shuffled randomly. In different simulations, the animats had 4 (figure panel A) or 8 (figure panel B) hidden nodes. The error bars are the standard errors for n = 12. PM  =  Expected average response for animats that do probability matching. Max  =  Expected average response for animats that perseverate. Random PM  =  Expected accuracy for animats that do probability matching in the random task. Random Max  =  Expected accuracy for animats that perseverate in the random task.

The results are shown in Figure 2 for both tasks (*pattern matching* and *random sequence*). An average response close to 0.67 indicates the animats did probability matching. When it is close to 1, it indicates they perseverated. To illustrate the range of strategies employed by animats, Table 1 shows sample responses from three individuals that belonged to the last generation.

**Table 1:** Example outcomes resulting from repetitive input patterns of length 3 and 729.

Pattern length	Task	Outcome	
3	Pattern matching	Input Sequence	10110110110110110110110110
		Animat’s Prediction	10110110110110110110110110
	Random sequence	Input Sequence	11111111001011110000111110
		Animat’s Prediction	10011101111111101101111011
729	Pattern matching	Input Sequence	01111111110011100111100011
		Animat’s Prediction	11111111111111111111111111
	Random sequence	Input Sequence	11011011011111110010011010
		Animat’s Prediction	11101111111111111111111111

Example of outcomes resulting from repetitive input patterns of length 3 and 729 under two task conditions: *Pattern matching* (when the animat evolved in an environment where it had to predict the next element of a sequence composed of a repetitive string of length 3 or 729), and *Random sequence* (when the animat, after evolving under a repetitive string of length 3 or 729, had to predict the next element of a completely random sequence). For sequences composed by short strings (3 digit long), the animat predicts all the elements correctly (pattern matching), but does probability matching when faced with the prediction of the next element in a shuffled random sequence. When the input sequence is composed of a very long repetitive string (729-digit long), the animat is not able to learn it, adopting a perseveration strategy, making many (expected) mistakes; but when the same animat has to predict the next element of a randomly shuffled sequence, it perseverates as well, achieving a better performance in comparison with the animats that had been able to learn a short-patterned sequence (3-digit long).

It can be observed that animats endowed with more hidden neurons and which evolved under input sequences composed of shorter repetitive strings were able to learn the repeating string and achieved a higher accuracy in the pattern matching task, approaching a 100% correct prediction rate. But when animats from the last generation performed the random sequence task, their accuracy was below the optimal value of 67% that they could have achieved if perseveration had been adopted; instead, their accuracy was close to the 56% expected for probability matching. Indeed, they matched the presentation probabilities of the digits in both tasks, but achieved quite different results – a nearly optimal accuracy in the pattern matching task and a sub-optimal one in the random sequence task.

At the other end of the spectrum, animats endowed with fewer hidden neurons and evolving under input sequences made up of longer repetitive strings relative to the processing power of their neural networks were not able to learn the repeating string. The animats showing the fittest behavior – and thus selected along successive generations – were those adopting a perseverating strategy, in which the outcome 1 should be predicted with a frequency close to 100%, reaching an accuracy slightly above 67%. When these animats were tested with a completely random sequence, they continued to perseverate and their accuracy remained around 67%, which is nearly optimal under these circumstances.

In short, the better an animat performs in the pattern matching task, the closer to probability matching it gets in the random sequence task. Animats that perseverate in the pattern matching task also perseverate in the random sequence task.

## Discussion

In the pattern matching task, animats that learn the pattern can always predict the next element of the sequence correctly – it is said that they “broke the code” presented to them by their environment. A superficial analysis of their behavior will lead to the conclusion that it behaves according to a “probability matching” strategy (which in fact it does, as a by-product of breaking the code), but with a performance that reaches optimality. An animat that is not able to learn the pattern is left with the next best strategy, perseveration, always choosing the most frequent outcome; in our experiment, this means that the next element of the sequence would be correctly predicted in two thirds of the trials, one third less than the animats that were able to learn the pattern achieved in accuracy.

**Figure 3:**
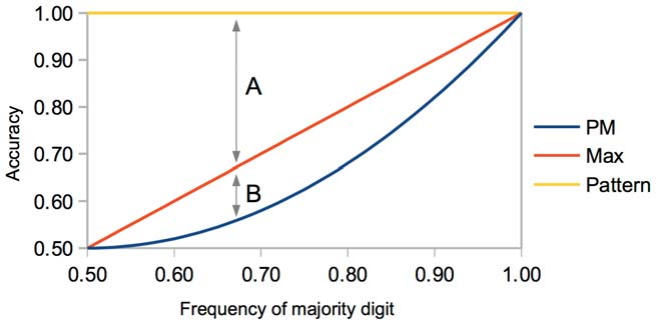
Accuracy for Different Strategies and Frequencies of Majority Digit in the Repeated Binary Choice Experiment. Predicted accuracy in the repeated binary choice experiment depending on the frequency of the majority digit and the employed strategy: *PM* (probability matching without pattern decoding), *Max* (perseveration) and *Pattern* (pattern decoding). For all digit frequencies except the frequency 1.0, the difference in accuracy between pattern decoding and perseveration (arrow A) is larger than the difference in accuracy between perseveration and probability matching without pattern decoding (arrow B).

In the present pattern matching task, the theoretical difference in accuracy between pattern decoding (leading to probability matching) and perseveration is one third, three times larger than the difference between probability matching and perseveration in the random sequence task, which is only one ninth. In fact, Figure 3 shows that, in a repeated binary choice experiment, for all frequencies of the most majority digit except the frequency 1.0, the difference in accuracy between pattern decoding and perseveration is larger than the difference in accuracy between perseveration and probability matching without pattern decoding. This result demonstrates that searching for patterns leads to larger gains when a pattern exists and to relatively smaller losses when it doesn’t. Thus, the ability to search for patterns would be maladaptive only in those scenarios where there are no structured patterns at all, this being just the case where very little can be predicted anyway, no matter what strategy one employs. In scenarios where structured patterns can be found, being able to search for and learn regular underlying patterns would be far more advantageous than perseveration.

An important feature of our simulations is that the resulting animats are experts in reproducing one particular pattern; unlike humans, they can’t generalize their knowledge to a larger set of patterns of the same complexity. It is possible, however, to consider the set of all animats with the same neural network structure, each trained to reproduce one of twelve patterns of a given length, as an individual having twelve networks that compete amongst themselves to generate a response. This individual would be capable of reproducing twelve different patterns, but it would still perform poorly with unstructured sequences, as those do not conform to any of the patterns it can recognize and do not contain any regularities to be extracted. In fact, even an individual that is able to reproduce a large number of complex patterns would perform poorly with unstructured sequences for the same reason – unstructured sequences do not conform to any of the patterns it can learn. It is also possible that an individual would be able to learn short periodic bit strings and at same time perform well when it detects that the bit string is approximately random. Although this possibility seems entirely plausible, given the present constraints, we do not believe that any neural network, no matter how complex, would detect that a bit string is approximately random if the network’s structure had evolved in an environment where all bit strings were made up of repeating patterns the network could learn. Distinguishing between a periodic sequence and an approximately random one is also rather complicated. At which point should one stop looking for patterns? What if one decides prematurely that a sequence is random when it is, in fact, periodic?

The animats also cannot generalize to other probability distributions. We have chosen this particular distribution (2/3 probability for the majority digit), because (1) it is close to values commonly used in experiments with human and biological animal subjects (around 0.7–0.8), (2) it is close enough to the 0.75 point, where the largest difference in performance between probability matching and perseveration occurs in the random sequence task, making the random task most relevant (Figure 2), (3) the selective pressure to break the pattern rather than perseverate is high (the difference in accuracy between the two strategies in the pattern matching task is 1/3, close to the maximum 1/2, when the probability is 0.5) and, most relevantly, (4) this probability value can be expressed as a bit string of length 3 (110, 101 and 011), which is the smallest pattern where the concept of “majority digit” applies. Other distributions, where the probability of the majority digit lies close to one of the end points of the 0.5–1.0 interval, are problematic. When the probability of the majority digit is close to 0.5, in the random sequence task, there is no difference in performance between probability matching and perseveration; in fact, all strategies are equivalent and therefore, if we had used such low values, no conclusions could have been drawn from having the animals perform the random sequence task, as all of them would have achieved the same level of performance no matter what strategy they employed. When the probability is close to 1, there is little difference in performance between pattern decoding, probability matching and perseveration in both the pattern matching and the random sequence tasks, and so no conclusions could have been drawn from such an experiment at all; there is little selective pressure to break the pattern in the pattern matching task, and we would expect our simulations to take more generations to arrive at the point where animals that can repeat the pattern, or to not arrive there at all, getting stuck at a local optimum where all animals perseverate.

In humans, pattern decoding may occur consciously or not. Like some animats, adult humans may never decide to perseverate, unless they are explicitly taught to. The intrinsic nature of probability matching may be illustrated by observing that people do probability matching when they engage in more ordinary tasks as well: determining the disease given its symptoms [16], classifying a height measurement as belonging to a man or a woman [23], taking the appropriate decision in response to alarms [24], localizing auditory-visual stimuli in space [25]. It has also been repeatedly pointed out that humans have difficulty in recognizing randomness and, as the gambler’s fallacy discussed in the introduction illustrates, have an incorrect intuition of it [3], [7].

In any case, humans are known for their ability to detect, in the surrounding environment, the existence of regularly recurring patterns that might be interpreted as the underlying structure of relevant events. If successful, one of the main benefits of this behavior is, in addition to reacting to sudden stimuli, being also able to predict upcoming changes in the environment and anticipate responses to them. Therefore, the ability to build models of the environment, according to model-based descriptions of reinforcement learning, appears to be a crucial evolutionary acquisition. In fact, Green and colleagues [26] show that a sub-optimal, probability matching, behavior can actually be observed in optimal Bayesian model-based learners, as long as they are initialized with biologically reasonable but incorrect beliefs about the underlying structure generating a sequence of events. Their main conclusion is that “human decision making is rational and model based and not consistent with model-free learning”.

However, the artificial life simulations presented here have suggested that it is not necessary to bring into play an issue of “rationality” versus “irrationality” in order to explain the non-optimal behavior associated to probability matching. A simpler and less involved explanation arises from the analysis of a plausible set of evolutionary constraints under which neural machinery responsible for prediction tasks has evolved. The present simulations employed very simple networks as model-free learners, bearing no prior beliefs. The non-optimality observed in the behavior of agents endowed with higher computational power, when confronted with poorly predictable sequences, results, according to our interpretation, from the discrepancy between the environment in which these agents evolved and the rather artificial task to which they were finally submitted. It has been observed that our cognition is subject to our need to survive in our daily lives, until we can generate descendants, and it may not perform optimally when the problem or the performance criterion isn’t ecologically relevant [27]. Therefore, if (i) humans were selected for behaving in a sufficiently patterned world and (ii) gradually acquired a neural machinery able to link a learned environmental pattern to successful actions, optimal actions should not be expected when coping with less structured, weakly predictable, environments. Under these possibly rarer and biologically less impacting circumstances, a sub-optimal performance – in comparison to other species – would be a fair toll to pay in exchange for a much higher fitness when surviving in a structured world.

In conclusion, an important reason why humans do not always maximize their expected utility is possibly that our brain is biased to make good decisions in the richer environment where we evolved, but poorer decisions in other, more artificial, situations. Although the human brain is flexible and can adapt to different environments in the short term, the strategies that helped our species survive in the long term also affect decision making today. The bias discussed here is likely to affect human behavior every time a sequence of observations is made, thus it may influence not just the repeated binary choice experiment and its variations, but also a wide range of experiments in decision-making and other branches of the cognitive sciences, as well as our daily lives.

## Methods

The simulation code, written in the Python and C++ programming languages, can be downloaded at http://www.fisio.icb.usp.br/~vinicius/downloads/probmatch.zip.

Artificial neurons were based on the perceptron model [22]. The output 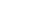 of neuron *j* at time-step *t* was determined according to equation 1:
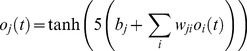
(1)where 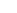 is the weight of the synapse between neuron j and neuron i and 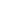 is neuron j’s bias. The activation function, f(x)  =  tanh(5x), yields a real number in the interval (−1, 1), therefore all outputs also belong to this interval. When the output was greater than or equal to 0, we considered that the animat had predicted the next element of the sequence to be 1, otherwise it had predicted 0.

The input node delivered feedback from the environment to the neural network. The feedback was positive (+5 input) if the animat had guessed correctly the previous element and negative (−5 input) otherwise.

The neural network architecture was feedforward with totally connected layers (Figure 1).

The synaptic weights could change through the Hebbian (or anti-Hebbian) learning rule: When two nodes fire at the same time, the synaptic weight between them increases (or decreases). When they fire at different times, the synaptic weight between them decreases (or increases). This was implemented according to equation 2:
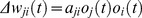
(2)Where 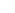 is the synapse’s learning parameter. When it is a positive number, the synapse is Hebbian, and when it is a negative number, the synapse is anti-Hebbian.

A chromosome was a set of genes representing all the synaptic weights, biases and learning parameters, which were real numbers in the interval [−1, 1). The initial genes at generation 0 were randomly generated with uniform distribution. One hundred chromosomes were generated in this manner and divided into 5 populations of 20 individuals. Neural networks were constructed based on these chromosomes and the animats performed the pattern matching task. The fitness number, i.e., the number of correct answers in the task, was calculated for every animat and used to select the parents of the next generation’s individuals. Selection occurred by tournament – two chromosomes were selected randomly within a population and the winner became a parent. Two parents were selected within a population to generate each child for that population in the next generation. Child chromosomes inherit their parents’ genes by crossover – for each gene, a parent was randomly selected and its gene was copied to the child chromosome – and mutation – with 5% probability, a number from the interval [−0.1, 0.1) was randomly generated with uniform distribution and added to the gene, but always keeping the gene in the interval [−1, 1). The populations evolved for 1000 generations, and at each 100 generations the fittest chromosome from each population migrated to a randomly chosen population, always keeping the number of individuals in each population at twenty.

The animats from the last generation performed the random sequence task in addition to the pattern matching task.

## References

[1] Vulkan N (2000). http://www.blackwell-synergy.com/links/doi/10.1111/1467-6419.00106.

[2] Hindmoor A (2006). Rational Choice..

[3] Barhillel M, Wagenaar W (1991). http://linkinghub.elsevier.com/retrieve/pii/019688589190029I.

[4] Parducci A, Polt J (1958). http://content.apa.org/journals/com/51/4/492.

[5] Behrend ER, Bitterman ME (1961). Probability-matching in the fish.. The American Journal of Psychology.

[6] Graf V, Bullock DH, Bitterman ME (1964). http://www.pubmedcentral.gov/articlerender.fcgi?artid=1404312.

[7] Falk R, Konold C (1997). http://doi.apa.org/getdoi.cfm?doi=10.1037/0033-295X.104.2.301.

[8] Sabes PN, Jordan MI (1996). https://exec.selfip.net/mirrors/bitsavers.org/pdf/mit/ai/aim/AIM-1568.pdf.

[9] Gaissmaier W, Schooler LJ (2008). http://www.ncbi.nlm.nih.gov/pubmed/19019351.

[10] Fantino E, Esfandiari A (2002). http://doi.apa.org/getdoi.cfm?doi=10.1037/h0087385.

[11] Unturbe J, Corominas J (2007). http://www.ncbi.nlm.nih.gov/pubmed/17784810.

[12] Wolford G, Miller MB, Gazzaniga MS (2000). The Left Hemisphere’s Role in Hypothesis Formation.. The Journal of Neuroscience.

[13] Miller MB, Valsangkar-Smyth M (2005). http://www.ncbi.nlm.nih.gov/pubmed/15708210.

[14] Wolford G, Newman SE, Miller MB, Wig GS (2004). http://www.ncbi.nlm.nih.gov/pubmed/15648726.

[15] Koehler DJ, James G (2009). http://www.ncbi.nlm.nih.gov/pubmed/19664762.

[16] Shanks DR, Tunney RJ, McCarthy JD (2002). http://doi.wiley.com/10.1002/bdm.413.

[17] Niv Y, Joel D, Meilijson I, Ruppin E (2002). http://adb.sagepub.com/cgi/doi/10.1177/10597123020101001.

[18] Hardy-Vallee B (2007). http://ieeexplore.ieee.org/lpdocs/epic03/wrapper.htm?arnumber=4218877.

[19] Seth AK (2001). http://adb.sagepub.com/cgi/doi/10.1177/105971230200900204.

[20] Seth AK (2007). http://www.pubmedcentral.nih.gov/articlerender.fcgi?artid=2440771&tool=pmcentrez&rendertype=abstract.

[21] Acuña DE, Schrater P (2010). http://www.pubmedcentral.nih.gov/articlerender.fcgi?artid=2996460&tool=pmcentrez&rendertype=abstract.

[22] Haykin S (1998). Neural Networks: A Comprehensive Foundation..

[23] Healy AF, Kubovy M (1981). http://content.apa.org/journals/xlm/7/5/344.

[24] Bliss JP, Gilson RD, Deaton JE (1995). http://www.class.uidaho.edu/psy562/Readings/Bliss%20et%20al%20(1995).pdf.

[25] Wozny DR, Beierholm UR, Shams L (2010). http://dx.plos.org/10.1371/journal.pcbi.1000871.

[26] Green CS, Benson C, Kersten D, Schrater P (2010). http://www.pubmedcentral.nih.gov/articlerender.fcgi?artid=2941269&tool=pmcentrez&rendertype=abstract.

[27] Haselton MG, Bryant GA, Wilke A, Frederick DA, Galperin A (2009). http://www.atypon-link.com/GPI/doi/abs/10.1521/soco.2009.27.5.733.

